# Endothelin-1 promotes epithelial–mesenchymal transition in human chondrosarcoma cells by repressing miR-300

**DOI:** 10.18632/oncotarget.11835

**Published:** 2016-09-02

**Authors:** Min-Huan Wu, Pei-Han Huang, Mingli Hsieh, Chun-Hao Tsai, Hsien-Te Chen, Chih-Hsin Tang

**Affiliations:** ^1^ Physical Education Office, Tunghai University, Taichung, Taiwan; ^2^ Sports Recreation and Health Management Continuing Studies, Tunghai University, Taichung, Taiwan; ^3^ Graduate Institute of Basic Medical Science, China Medical University, Taichung, Taiwan; ^4^ Department of Life Science, Tunghai University, Taichung, Taiwan; ^5^ Department of Orthopedic Surgery, China Medical University Hospital, Taichung, Taiwan; ^6^ School of Medicine, China Medical University, Taichung, Taiwan; ^7^ School of Chinese Medicine, China Medical University, Taichung, Taiwan; ^8^ Department of Biotechnology, College of Health Science, Asia University, Taichung, Taiwan

**Keywords:** Endothelin-1, epithelial to mesenchymal transition, AMPK, Twist, miR-300

## Abstract

Chondrosarcoma is a malignant tumor of mesenchymal origin predominantly composed of cartilage-producing cells. This type of bone cancer is extremely resistant to radiotherapy and chemotherapy. Surgical resection is the primary treatment, but is often difficult and not always practical for metastatic disease, so more effective treatments are needed. In particular, it would be helpful to identify molecular markers as targets for therapeutic intervention. Endothelin-1 (ET-1), a potent vasoconstrictor, has been shown to enhance chondrosarcoma angiogenesis and metastasis. We report that ET-1 promotes epithelial–mesenchymal transition (EMT) in human chondrosarcoma cells. EMT is a key pathological event in cancer progression, during which epithelial cells lose their junctions and apical-basal polarity and adopt an invasive phenotype. Our study verifies that ET-1 induces the EMT phenotype in chondrosarcoma cells via the AMP-activated protein kinase (AMPK) pathway. In addition, we show that ET-1 increases EMT by repressing miR-300, which plays an important role in EMT-enhanced tumor metastasis. We also show that miR-300 directly targets Twist, which in turn results in a negative regulation of EMT. We found a highly positive correlation between ET-1 and Twist expression levels as well as tumor stage in chondrosarcoma patient specimens. Therefore, ET-1 may represent a potential novel molecular therapeutic target in chondrosarcoma metastasis.

## INTRODUCTION

Chondrosarcoma, the second most common type of bone cancer, is a heterogeneous group of malignancies that are characterized by the production of cartilage matrix. Chondrosarcomas can be classified into three histologic grades: grade I (low-grade), grade II (intermediate grade) or grade III (high-grade). The higher the grade, the more likely the tumor is to metastasize to other areas of the body. Although high-grade tumors develop in approximately only 5–10% of chondrosarcoma patients, these aggressive tumors remain the major cause of death [1, 2]. Thus, metastasis is a major obstacle that must be overcome for the successful treatment of chondrosarcoma. Exploring the molecular basis of metastasis may help to improve the early detection, prevention, intervention, and prognostic evaluation of a chondrosarcoma.

Secreted proteins are responsible for crosstalk among cancer cells and may facilitate the progression of metastasis, particularly within the steps of epithelial–mesenchymal transition (EMT), migration, and invasion [3–6]. Endothelin-1 (ET-1) is a potent vasoconstrictor and the most abundantly and widely expressed member of the endothelin family of proteins (ET-1, ET-2, and ET-3). Aberrant ET-1 is implicated in the pathobiology of a wide range of human tumors [7]. ET-1 acts as a survival factor from apoptosis via the endothelin A receptor (ET_A_R) [8] or ET_B_R [9] in an autocrine/paracrine manner in several different types of tumor cells. It has been reported that an association between ET-1 and various secreted factors or matrix proteins plays an important role in tumor progression and metastasis [10], while other research has demonstrated that the ET-1/ET_A_R autocrine pathway drives EMT in ovarian tumor cells by inducing an invasive phenotype [11]. These findings suggest that ET-1 induces the EMT process and may represent a novel target for therapeutic intervention in tumor angiogenesis and metastasis.

The metastatic process consists of distinct steps, including tumor growth, angiogenesis, tumor cell detachment, EMT, survival within blood and lymphatic vessels and embolization, extravasation, mesenchymal–epithelial transition (MET), formation of micrometastasis and, finally, growth of macrometastasis. EMT increases the metastatic and invasive potential of these cells. Downregulation of epithelial markers such as cytokeratin and E-cadherin and upregulation of mesenchymal markers such as vimentin, N-cadherin characterize the EMT process. Usually, inhibition of E-cadherin expression leads to induction of N-cadherin expression, which has been associated with tumor invasiveness [6]. Twist, and other transcription factors such as TGF-β, Snail, Slug and Sip1, have been show to play a regulatory role in EMT [6].

MicroRNAs (miRNAs) are small, endogenous, evolutionarily conserved non-coding ribonucleotide acids. It is estimated that up to 3% of the human genome codes for miRNA sequences. MiRNAs are involved in numerous biological processes, including cell growth, development, differentiation, proliferation, and death. They bind to complementary sequences in the 3′ untranslated regions (3′ UTRs) of their target mRNAs, resulting in degradation or blocking of gene translation. Studies have demonstrated that miRNAs modulate the metastatic process in many tumors [12]. Recently, miRNA microarray analysis has highlighted differential expression of miRNAs between mesenchymal-like cancer cells and epithelial-like cancer cells [13, 14]. Remarkably, miR-300 was down-regulated in cancer cells that underwent EMT compared with miR-300 expression in typical epithelial phenotype carcinoma cells, indicating that miR-300 may affect EMT. This study found that ET-1 promotes EMT in chondrosarcomas by inhibiting miR-300 via the AMP-activated protein kinase (AMPK) signaling pathways. This work provides a novel insight into the mechanism of ET-1 in metastasis of human chondrosarcoma cells.

## RESULTS

### ET-1 promotes EMT in human chondrosarcoma cells

**Figure 1:**
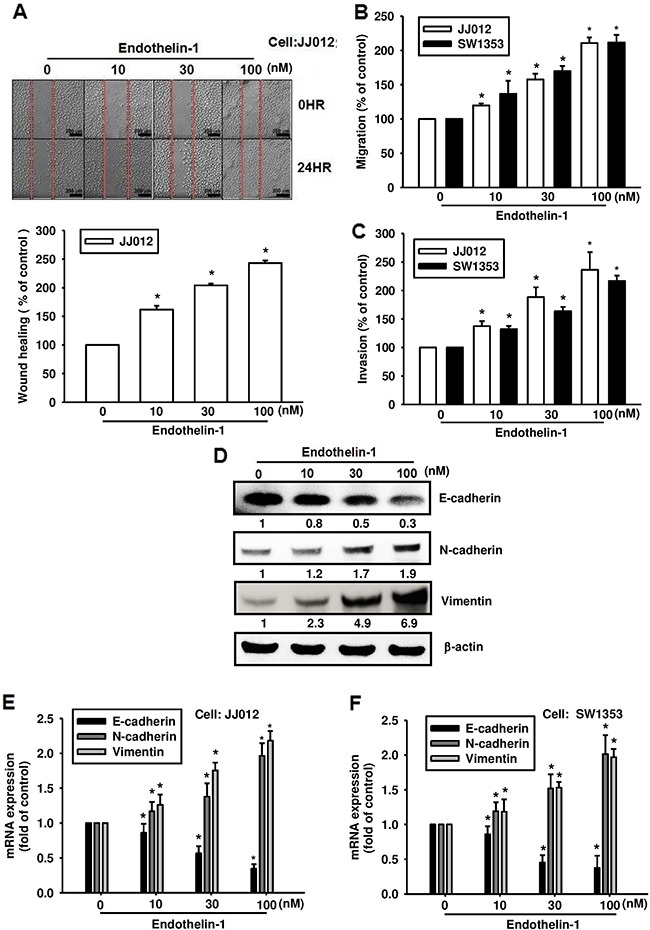
ET-1 promotes cell migration and EMT in chondrosarcoma cells Cells were incubated with ET-1 (10~100 nM) for 24 h, and cell migration was measured by the wound-healing assay **A.** (Scar bar = 200 μm), the Transwell assay **B.** and the invasion assay **C.** (n=4-6). **D-F.** The cells were incubated with ET-1 for 24 h, and the protein and mRNA expression of E-cadherin, N-cadherin and vimentin were measured by western blot (D) and qPCR (E&F) (n=6-8). Results are expressed as the mean ± S.E.M. *p < 0.05 compared with control.

ET-1 has been implicated in the angiogenesis and metastasis of human chondrosarcoma cells [15, 16]. To investigate the effects of ET-1 on chondrosarcoma cell migration, JJ012 and SW1353 cells were treated with different concentrations of ET-1. As shown in Figure 1A-1C, ET-1 induced wound healing (Figure 1A), migration (Figure 1B) and invasion (Figure 1C) of chondrosarcoma cells in a dose-dependent manner. The essential features of EMT in the context of tumor progression are enhanced cell migration and invasion [17, 18]. To examine whether ET-1 is required for EMT in chondrosarcoma, the chondrosarcoma cell lines were treated with ET-1. Induction of EMT after ET-1 treatment was demonstrated by a shift from the expression of an epithelial marker (E-cadherin) to mesenchymal markers (N-cadherin and vimentin) (Figure 1D-1F).

**Figure 2:**
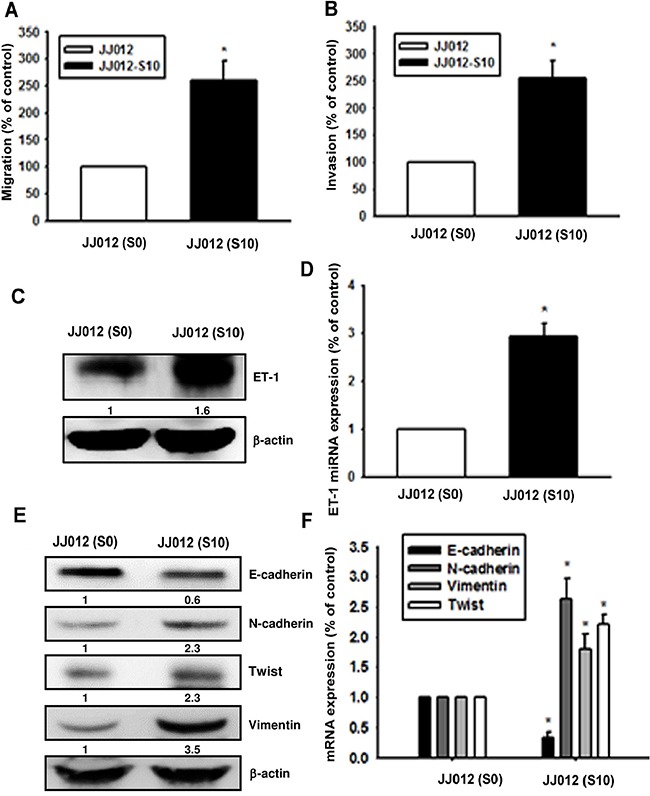
Upregulation of ET-1 and EMT in migration-prone chondrosarcoma cells After 10 rounds of selection using the cell culture insert system, the migration-prone subline JJ012(S10) exhibited greater migration **A.** and invasion ability **B.** than the original JJ012(S0) line. **C-F.** ET-1 and EMT marker expression in JJ012(S0) and JJ012(S10) were exmained by western blot and qPCR (n=3). Results are expressed as the mean ± S.E.M. * *p*< 0.05 compared with JJ012(S0).

To further clarify whether ET-1 is associated with migration activity and EMT in chondrosarcoma, highly migratory JJ012(S10) cells were selected by Transwell assay. Results revealed that JJ012(S10) cells show higher migration (Figure 2A) and invasion abilities (Figure 2B) as well higher expression of ET-1 and EMT markers (N-cadherin, vimentin and Twist) as compared with JJ012(S0) cells (Figure 2C-2F). Moreover, E-cadherin levels were reduced in JJ012(S10) cells as compared with JJ012(S0) cells (Figure 2E & 2F). The findings indicate that ET-1 promotes EMT in chondrosarcoma cells.

### ETRs are involved in ET-1-induced EMT in chondrosarcoma

**Figure 3:**
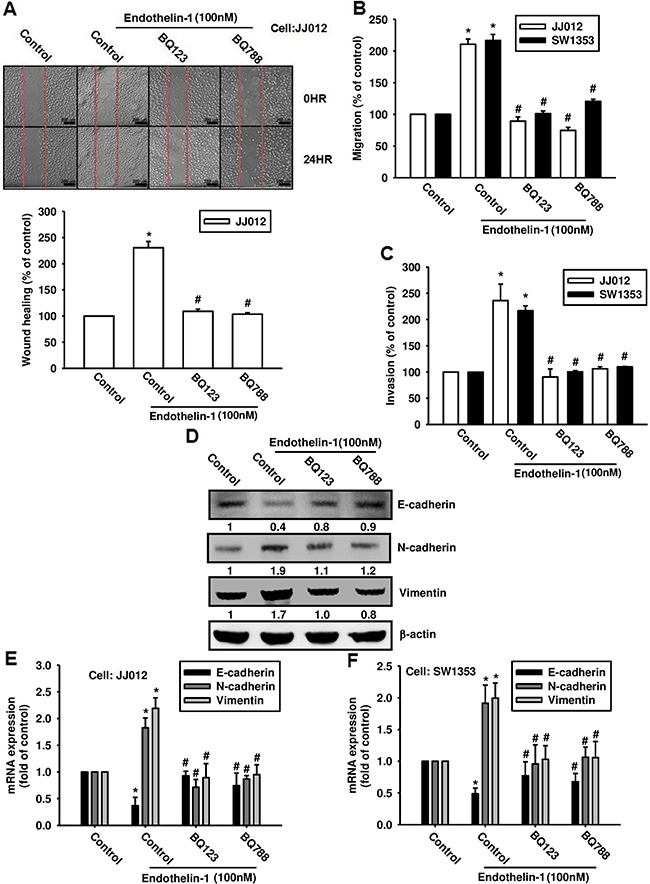
ET-1 promotes cell migration and EMT through ETRs **A & B.** Cells were incubated with BQ123 (10 μM) or BQ788 (10 μM) for 30 mins before undergoing stimulation with ET-1 (100 nM) for 24 h. Cell migration was measured by the wound-healing assay (A) (Scar bar = 200 μm), the Transwell assay (B) and invasion assay **C.** (n=4-5). **D-F.** Cells were incubated with BQ123 (10 μM) or BQ788 (10 μM) for 30 mins, then treated with ET-1 for 24 h. Protein and mRNA expression levels of E-cadherin, N-cadherin and vimentin were measured by western blot (D) and qPCR (E&F) (n=5-6). Results are expressed as the mean ± S.E.M. * *p*< 0.05 compared with control. # *p* < 0.05 compared with the ET-1-treated group.

ET-1 acts through two distinct subtypes of G-protein coupled receptors (i.e., ET_A_ and ET_B_) [19, 20]. Therefore, we hypothesized that the ET receptors may be involved in ET-1-induced EMT and cell migration in chondrosarcoma. Pretreatment of chondrosarcoma cells with the ET_A_R antagonist BQ123 and the ET_B_R antagonist BQ788 abolished ET-1-induced wound healing (Figure 3A), migration (Figure 3B), and invasion (Figure 3C). We further examined whether ET-1 has the ability to trigger activation of EMT-related markers via the ETRs. Our results show that ETR inhibitors reverse ET-1-induced changes in expression of EMT markers (Figure 3D-3F). These data suggest that ET-1 promotes EMT in chondrosarcoma via the ETRs.

### Twist is required for ET-1-increased EMT and cell migration in human chondrosarcoma cells

**Figure 4:**
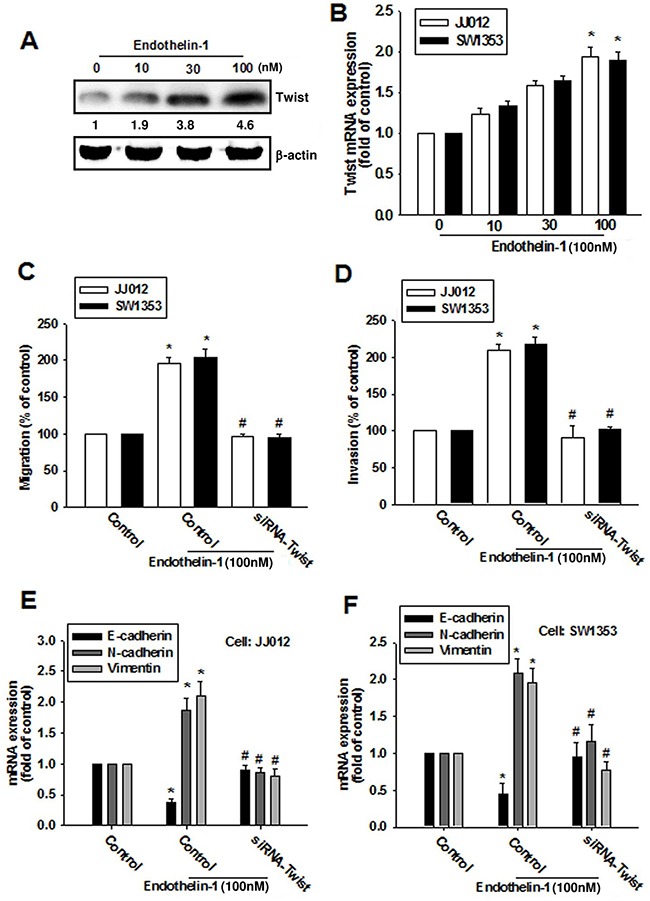
ET-1 enhances cell migration and EMT through Twist activation **A & B.** Cells were incubated with ET-1 (10~100 nM) for 24 h, and the protein and mRNA expression levels of Twist were measured by western blot (A) and qPCR (B). **C-F.** Cells were transfected with Twist siRNA for 24 h followed by stimulation with ET-1 (100 nM) for 24 h (n=4-5). Cell migration and EMT marker expression were examined by Transwell migration assay, invasion assay and qPCR. Results are expressed as the mean ± S.E.M. *p< 0.05 compared with control. #*p* < 0.05 compared with the ET-1-treated group.

Previous studies have indicated that Twist promotes the initiation of EMT [21, 22]. We therefore hypothesized that Twist may be involved in ET-1-increased EMT and cell migration in human chondrosarcoma cells. Treatment of cells with ET-1 enhanced Twist expression in a dose-dependent manner (Figure 4A & 4B). To further evaluate whether the activation of Twist is required for ET-1-induced migration and EMT, cells were transiently transfected with Twist siRNA, before undergoing ET-1 stimulation. The results revealed that ET-1 elicits a significant change in cell migration (Figure 4C) and invasion (Figure 4D) as well as EMT (Figure 4E & 4F), all of which were drastically attenuated in the presence of Twist siRNA. Twist therefore plays a critical role in ET-1-induced EMT and cell migration.

### The AMPK signaling pathway is involved in ET-1-induced EMT and cell migration

**Figure 5:**
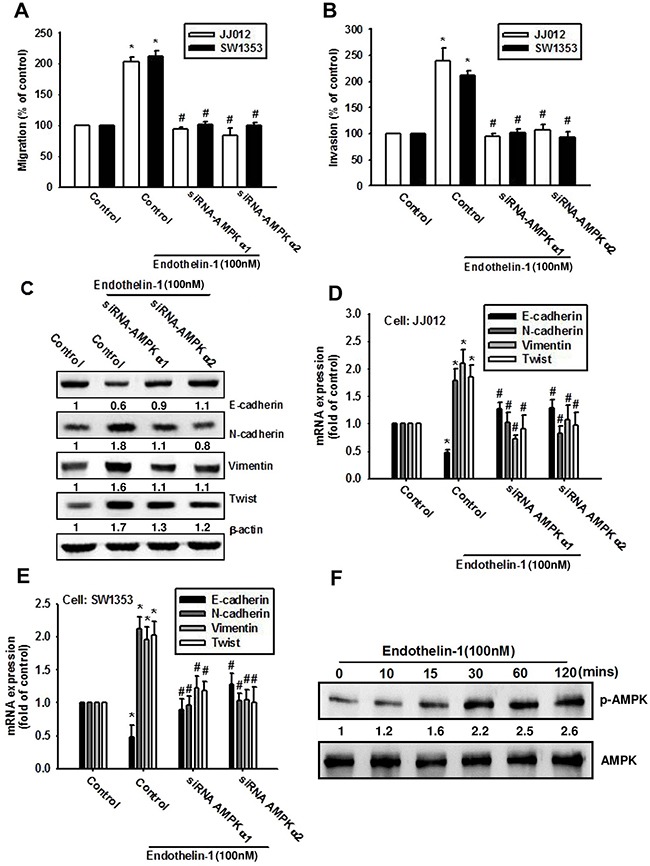
AMPK is involved in ET-1-induced EMT and cell migration **A-E.** Cells were transfected with AMPKα1 or AMPKα2 siRNA for 24 h, then stimulated with ET-1 for 24 h. Cell migration (A), invasion (B) and EMT marker expression (C-E) were measured by Transwell migration assay, invasion assay, western bolt and qPCR. JJ012 cells were treated with ET-1, the AMPK phosphorylation was examined by western blot **F.** (n=4-5). Results are expressed as the mean ± S.E.M. * *p*< 0.05 compared with control. #*p* < 0.05 compared with the ET-1-treated group.

AMPK has been shown to regulate human chondrosarcoma metastasis [23, 24]. We therefore investigated whether AMPK mediates ET-1-induced EMT and migration of chondrosarcoma cells. Transfection of chondrosarcoma cells with AMPK-specific siRNA (AMPKα1 or AMPKα2 siRNA) abolished ET-1-induced cell migration (Figure 5A) and invasion (Figure 5B). Moreover, AMPK-specific siRNA reversed ET-1-induced EMT (Figure 5C-5E). Subsequently, we directly measured AMPK phosphorylation in response to ET-1 and found that stimulation of cells with ET-1 increased phosphorylation of AMPK in a time-dependent manner (Figure 5F). These data suggest that AMPK activation is involved in ET-1-induced cell migration and EMT in human chondrosarcomas.

### ET-1 induces Twist expression by inhibiting miR-300 in chondrosarcomas

**Figure 6:**
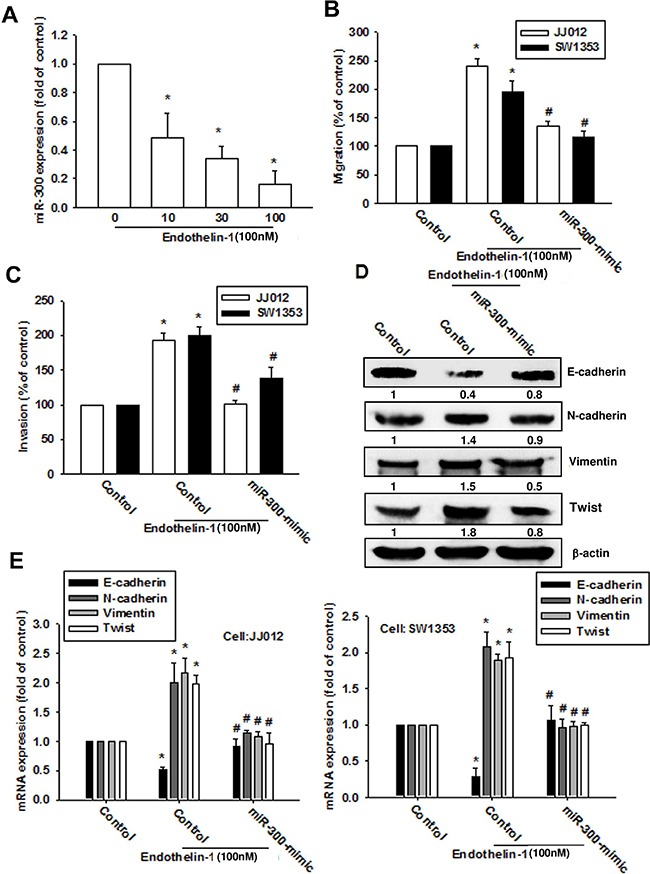
ET-1 promotes cell migration and EMT expression by downregulating miR-300 expression **A.** Cells were incubated with ET-1 (10~100 nM) for 24 h, and miR-300 expression was examined by q-PCR. Cells were transfected with a control miRNA or miR-300 mimic for 24 h then stimulated with ET-1 (100 nM) for 24 h. **B-E.** Cell migration (B), invasion (C) and EMT marker expression (D-E) were examined by Transwell migration assay, invasion assay, western blot and q-PCR (n=4-6). Results are expressed as the mean ± S.E.M. * *p*< 0.05 compared with control. #*p* < 0.05 compared with the ET-1-treated group.

Recent evidence has highlighted the role played by miRNAs in modulating the metastatic process in solid tumors [25]. Many studies have subsequently been conducted and a large number of miRNAs have been found to correlate with the EMT process [26]. We next used 3 online computational algorithms (TargetScan, miRanda and miRWalk) to explore candidate miRNAs that target Twist. The results indicate that miR-300 targets the 3'-untranslated region (UTR) segment of Twist mRNA. We found that miR-300 expression was decreased in a dose-dependent manner after ET-1 treatment (Figure 6A). When we transfected chondrosarcomas with a miR-300 mimic then treated them with ET-1, the miR-300 mimic but not the control miRNA abolished ET-1-induced Twist expression and EMT (Figure 6D-6E). We also confirmed the role of miR-300 in cell migration by targeting Twist. The data indicate that the miR-300 mimic inhibited ET-1-induced migration (Figure 6B) and invasion (Figure 6C).

**Figure 7:**
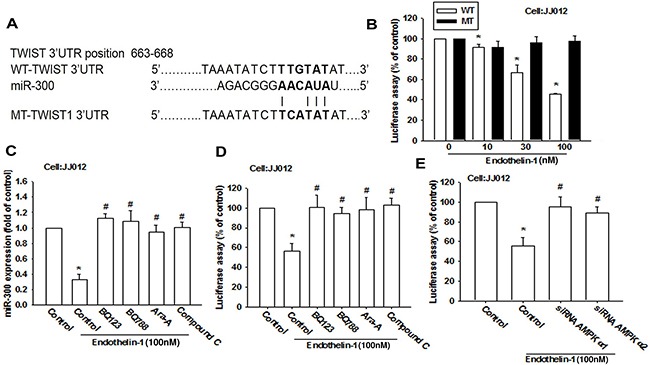
miR-300 directly represses Twist expression via binding to the 3'UTR of the human Twist **A.** Schematic 3'UTR representation of the human Twist containing the miR-300 binding site. **B.** Cells were transfected with a wt or mutant Twist 3′UTR luciferase plasmid for 24 h followed by stimulation with ET-1 (10~100 nM) for 24 h, and the relative luciferase activity was measured. **C-E.** Cells were pretreated with BQ123 (10 μM), BQ788 (10 μM), Ara A (1 mM) and compound C (10 μM) for 30 min or pre-transfected with specific siRNAs for 24 h followed by stimulation with ET-1 for 24 h. miR-300 expression or wild-type Twist 3′UTR luciferase activity were examined (n=4-5). Results are expressed as the mean ± S.E.M. * *p*< 0.05 compared with control. #*p* < 0.05 compared with the ET-1-treated group.

To elucidate whether miR-300 specifically targets the Twist 3′UTR, we constructed luciferase reporter vectors harboring wild-type 3′UTR of the Twist mRNA (WT-Twist-3′UTR) and mismatches in the predicted miR-300 binding site (MT-Twist-3′UTR; Figure 7A). These vectors were then transfected into JJ012 cells after undergoing treatment with various concentrations of ET-1. As shown in Figure 7B, ET-1 decreased luciferase activity in the WT-Twist-3′UTR plasmid but not in the MT-Twist-3′UTR, indicating that miR-300 directly represses Twist protein expression via binding to the 3′UTR of human Twist. In addition, BQ123, BQ788 and AMPK inhibitors (Ara A and compound C) reversed ET-1-inhibited miR-300 expression (Figure 7C) and WT-Twist-3′UTR luciferase activity (Figure 7D). Furthermore, AMPKα1 or α2 siRNA also reversed ET-1-inhibited WT-Twist-3′UTR luciferase activity (Figure 7E). These data indicate that miR-300 directly represses Twist expression via binding to the 3′UTR of human Twist through ETRs and AMPK signaling.

### ET-1 expression is positively correlated with Twist expression in resected chondrosarcoma specimens

**Figure 8:**
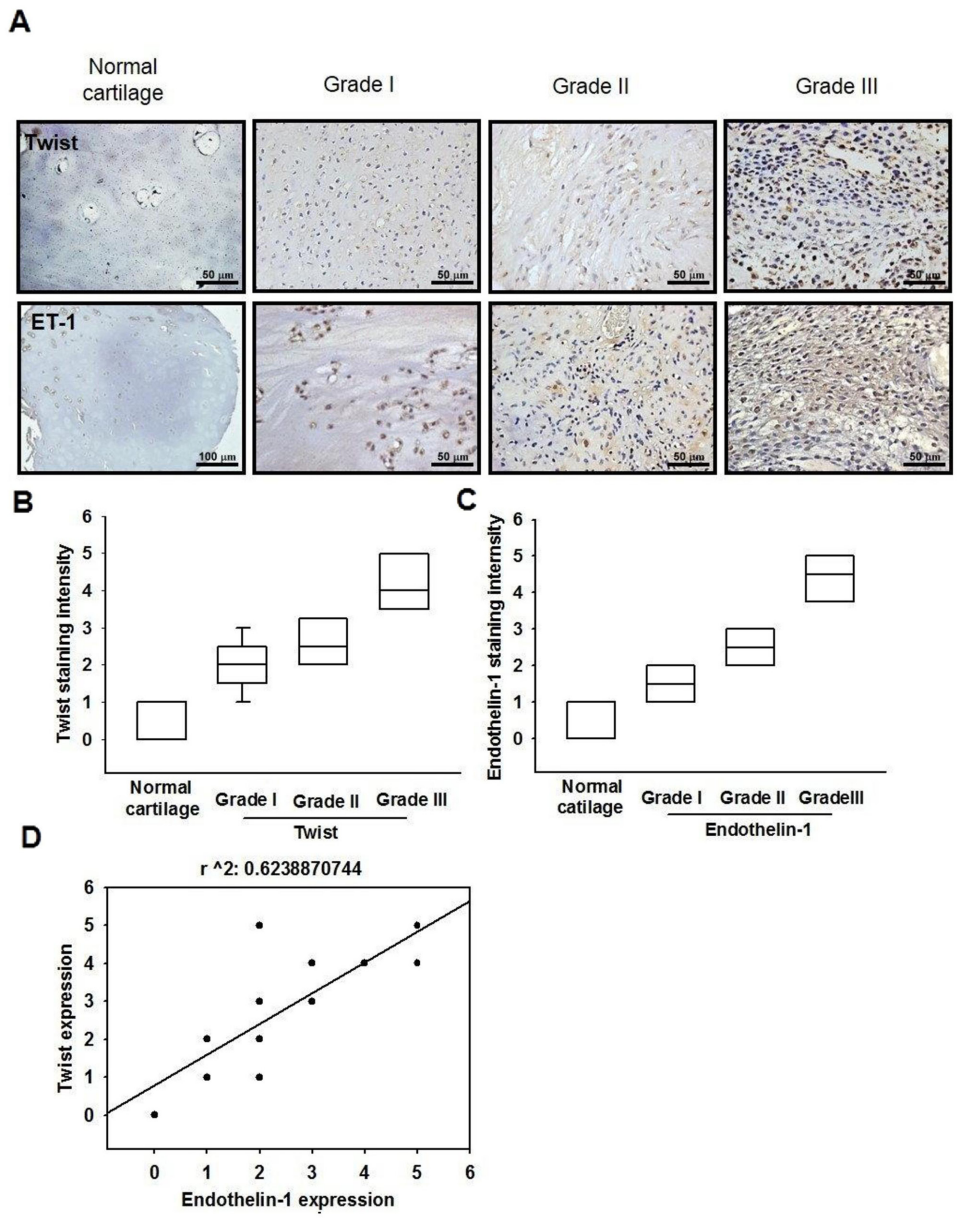
The correlation of ET-1, Twist and tumor stages in human chondrosarcoma tissues **A.** IHC analysis of ET-1 and Twist expression in normal cartilage and chondrosarcoma tissues (Scar bar = 50 μm). Quantitative data are shown in **B & C.** Correlation and quantitative data are shown in **D.** Data are expressed as the mean ± SEM.

To determine the clinical significance of ET-1 and Twist in patients with chondrosarcoma, we performed an immunohistochemical (IHC) assay using a tissue microarray to compare the expression of ET-1 and Twist in normal cartilage and different grades of chondrosarcomas. Representative examples of IHC staining for ET-1 and Twist in normal cartilage and histopathologically different grades of chondrosarcoma tissues are shown in Figure 8A. The expression of ET-1 and Twist increased significantly with tumor progression (Figure 8B & 8C). In addition, Pearson's correlation revealed significantly positive correlations between ET-1 expression and Twist (*r*^2^ = 0.6238, *P* < 0.0001) (Figure 8D). Higher levels of ET-1 expression were found in tumor specimens and were positively correlated with Twist expression in chondrosarcomas.

## DISCUSSION

The advent of effective systemic chemotherapy has dramatically improved long-term survival in other primary malignant bone tumors, such as osteosarcoma and Ewing's sarcoma, chondrosarcoma continues to have a poor prognosis due to the limited effectiveness of adjuvant therapy [1]. Chondrosarcoma shows a predilection for metastasis to the lungs. Much has been learned about the commonly known and well-studied process of EMT during the malignant progression to chondrosarcoma. It is therefore important to explore potential targets for preventing the occurrence of EMT in chondrosarcoma. This study describes how ET-1 enhances the expression of Twist in human chondrosarcoma cells and subsequently increases EMT and metastasis. In addition, the downregulation of miR-300 through the ETRs and AMPK pathway is mediated by ET-1-induced EMT and tumor metastasis.

Considerable evidence suggests that tumor cells expressing aberrant levels of ET-1 facilitate tumor development and progression. Our previous study indicated that ET-1 facilitates oncogenesis in human chondrosarcoma by increasing cell migration via the matrix-metalloproteinase (MMP) family and cyclooxygenase (COX)-2 overexpression [15, 16]. We have also previously demonstrated that ET-1 facilitates tumor metastasis and tumorigenesis by mediating angiogenesis in human chondrosarcomas [27]. However, the molecular mechanisms of ET-1-regulated EMT in human chondrosarcomas are not well characterized. The present study reveals that ET-1 signaling has a distinct function in chondrosarcoma, namely, regulation of EMT and cell migration.

Higher AMPK expression has been found to correlate with lower tumor grade and/or grade in various cancers, including ovarian, hepatocellular, pancreatic, breast, and gallbladder cancers [28, 29], and evidence indicates that the tumors in Peutz-Jeghers syndrome may result from deficient activation of AMPK due to inactivation of serine/threonine kinase 11, the major upstream kinase required for AMPK activation [30]. Recently, several studies have shown that AMPK plays an important role in metastasis through its effects on cell migration, and that AMPK stimulates cell motility via microtubule polymerization [31]. Silencing AMPK expression disrupts front-rear polarity and results in directional migration defects [32–34]. Our results indicate that ET-1 induces cell migration by activation of AMPK in human chondrosarcoma cells. AMPK may therefore represent a potential target for the development of new anticancer drugs, in particular those targeting metastasis.

miRNAs control gene expression by binding to complementary sequences in 3′UTRs of target mRNAs [35, 36]. Deregulated miRNA expression has been cited in human cancers and may affect multiple steps during metastasis [37]. In particular, the following miRNAs can regulate metastatic ability in osteosarcoma: miR-507 [38], miR-497 [39], miR-519d [23], miR-185 [40], miR-218 [40] and miR-200b [41]. Our study indicates that miR-300 is downregulated in response to ET-1; miR-300 reportedly suppresses tumor formation in human glioblastoma, making it an attractive candidate biomarker for the prediction of response to cancer treatment [42, 43]. In this study, transfection of cells with miR-300 mimic reduced ET-1-induced cell migration, indicating that miR-300 can function as a tumor suppressor.

Previous research has demonstrated Twist functions in many stages of cancer metastasis [44, 45]. ET-1 has been reported to regulate Twist expression [46]. Our study found increasing Twist mRNA and protein expression in JJ012/S10 cells. Knockdown of Twist expression via transfection with Twist siRNA abolished ET-1-induced migration activity of chondrosarcoma cells, demonstrating that Twist is involved in ET-1-mediated cell migration. miRNA target prediction analysis proved that Twist is the targets of miR-300; transfection of cells with the miR-300-mimic strongly inhibited ET-1-induced Twist expression. Our findings indicate that miR-300 directly represses Twist protein expression through binding to the 3′UTR of human Twist genes, and thus negatively regulates Twist-mediated metastasis.

In conclusion, metastasis plays a critical role in the progression of tumors and is the main cause of death from cancer. Chemotherapy and radiation play limited roles in primary treatment of chondrosarcoma and no specific standardized therapy has as yet proven to be effective for chondrosarcoma [47]. Our study elucidates the mechanism of ET-1-induced EMT in chondrosarcoma; miR-300 may play a pivotal role in this process. Our findings provide a novel insight into the role of ET-1 in cancer metastasis and indicate that ET-1 may be a novel therapeutic target in chondrosarcoma metastasis.

## MATERIALS AND METHODS

### Materials

Protein A/G beads, anti-mouse and anti-rabbit IgG-conjugated horseradish peroxidase, rabbit polyclonal antibodies specific for vimentin, N-cadherin, E-cadherin, Twist, p-AMPK, AMPK, and β-actin were purchased from Santa Cruz Biotechnology (Santa Cruz, CA, USA). ON-TARGETplus siRNAs of Twist, AMPKα1, AMPKα2 and control were purchased from Dharmacon Research (Lafayette, CO, USA). Recombinant human ET-1 was purchased from PeproTech (Rocky Hill, NJ, USA). miRNA control and miR-300 mimic were purchased from Invitrogen (Carlsbad, CA, USA). All other chemicals were obtained from Sigma–Aldrich (St Louis, MO, USA).

### Cell culture

The human chondrosarcoma cell line (JJ012) was kindly provided by the laboratory of Dr. Sean P Scully (University of Miami School of Medicine, Miami, FL). The human chondrosarcoma cell line (SW1353) was obtained from the American Type Culture Collection. Cells were cultured in Dulbecco's modified Eagle's medium (DMEM)/α-MEM supplemented with 10% fetal bovine serum and 100 units/mL penicillin/streptomycin at 37°C in a humidified chamber in 5% CO_2_. The basal levels of ET-1 in JJ012 and SW1353 cells are 2.16 pg/ml and 1.33 pg/ml, respectively.

### Western blot analysis

The cellular lysates were prepared and proteins were then resolved on SDS–PAGE and transferred to Immobilon polyvinyldifluoride (PVDF) membranes. The blots were blocked with 4% BSA for 1 h at room temperature and then probed with rabbit anti-human antibodies against AMPK, p-AMPK, E-cadherin, N-cadherin, vimentin or Twist (1:1000) for 1 h at room temperature. After three washes, the blots were incubated with a donkey anti-rabbit peroxidase-conjugated secondary antibody (1:1000) for 1 h at room temperature. The protein bands were visualized by enhanced chemiluminescence using ImageQuant LAS 4000 (GE Healthcare Life Sciences, Little Chalfont, UK). Quantitative data were obtained using a computing densitometer and ImageQuant software (Molecular Dynamics, Sunnyvale, CA).

### Quantitative real time PCR

The quantitative real-time PCR (qPCR) analysis was carried out using the Taqman® one-step PCR Master Mix (Applied Biosystems, Foster City CA). 100 ng of total cDNA was added per 25 μl reaction with sequence-specific primers and Taqman® probes. Sequences for all target gene primers and probes were purchased commercially (β-actin was used as the internal control) (Applied Biosystems, CA). Quantitative RT-PCR assays were carried out in triplicate on a StepOnePlus sequence detection system. The cycling conditions were 10 min polymerase activation at 95°C followed by 40 cycles at 95°C for 15 sec and 60°C for 60 sec. The threshold was set above the non-template control background and within the linear phase of target gene amplification to calculate the cycle number at which the transcript was detected (denoted as CT).

### miRNA qPCR analysis

Total RNAs were extracted and cDNA was synthesized using the Mir-X™ miRNA First-Strand Synthesis Kit (Clontech, CA, USA). Quantitative RT-PCR assays were carried out in triplicate on a StepOnePlus sequence detection system. The cycling conditions were 10 min polymerase activation at 95°C followed by 40 cycles at 95°C for 15 sec and 60°C for 60 sec. Relative gene expression was quantified using an endogenous control gene (U6). The threshold cycle (CT) was defined as the fractional cycle number at which fluorescence passed a fixed threshold, and relative expression was calculated using the comparative CT method.

### Transwell migration and invasion assays

All cell migration assays were performed using Transwell inserts (8-μm pore size; Costar, NY) in 24-well dishes. Chondrosarcoma cells were pretreated for 30 min with the indicated concentrations of inhibitors or vehicle (0.1% DMSO). Cells (1 × 10^4^ in 200 μl of serum-free medium) were seeded in the upper chamber of the Transwell and 300 μl of the same medium containing varying concentrations of ET-1 was placed in the lower chamber. Each experiment was performed with triplicate wells and repeated at least 3 times. For the cell invasion assay, each well was pre-coated with Matrigel (25 mg/50 mL; BD Biosciences, Bedford, MA) to form a continuous, thin layer. Protocol was followed in the migration assay.

### Establishment of migration-prone sublines

Subpopulations of JJ012 cells were selected according to their differential migration abilities using the cell culture insert system as described above. After overnight migration, cells that penetrated through pores and migrated to the undersides of filters were trypsinized and harvested for a second round of selection. After 10 rounds of selection, migration-prone subline was designated as JJ012 (S10). Original cells were designated as JJ012 (S0) [48].

### Wound healing assay

For wound-healing migration assays, cells were seeded on 12-well plates at a density of 1 × 10^5^ cells/well in culture medium. At 24 h after seeding, the confluent monolayer of culture was scratched with a fine pipette tip, and migration was visualized by microscopy. The rate of wound closure was observed at the indicated times.

### Plasmid construction and luciferase reporter assay

Wild-type (wt) Twist-3'-UTR was constructed into the pGL2-Control vector. The mutation of Twist-3'-UTR was performed by Quickchange™ site-directed mutagenesis protocol (Stratagene; La Jolla, CA, USA), according to the manufacturer's instructions.

To analysis 3'-UTR luciferase activity, JJ012 cells were transfected with wt-Twist-3'UTR or mutant (mt)-Twist-3'UTR luciferase plasmids. Cells were lysated after 24 h of transfection, harvested and detected using a luciferase assay system (Promega; Madison, WI, USA).

### Immunohistochemistry analysis

The human chondrosarcoma tissue array was purchased from Biomax (Rockville, MD, USA; 6 cases for normal cartilage, 24 cases for grade I chondrosarcoma, 9 cases for grade II chondrosarcoma, and 15 cases for grade III chondrosarcoma). Fixed and paraffin-embedded tissues were deparaffinized with xylene, and rehydrated through a graded series of alcohols to water. Endogenous peroxidase activity was blocked with 3% hydrogen peroxide. Heat-induced antigen retrieval was carried out for all sections in 0.01 M sodium citrate buffer, pH 6 at 95°C for 20 min. Human ET-1 or Twist antibodies were applied at a dilution of 1:200 and incubated at 4°C overnight. Bound antibodies were detected by NovoLink Polymer Detection System (Leica Microsystems, Newcastle, UK) and visualized with the diaminobenzidine reaction. The sections were counterstained with hematoxylin. The staining intensity was evaluated as 0, 1+, 2+, 3+, 4+, and 5+ for no staining, very weak staining, weak staining, moderate staining, strong staining, and very strong staining, respectively, by two independent and blinded observers. IHC score was determined as the sum of the intensity score.

### Statistics

All data are presented as the mean ± SEM. Statistical comparison of two groups was performed using the Student's t-test. Statistical comparisons of more than two groups were performed using one-way analysis of variance with Bonferroni's post hoc test. In all cases, *P* < 0.05 was considered significant.
